# Surface expression, single-channel analysis and membrane topology of recombinant *Chlamydia trachomatis *Major Outer Membrane Protein

**DOI:** 10.1186/1471-2180-5-5

**Published:** 2005-01-26

**Authors:** Heather E Findlay, Heather McClafferty, Richard H Ashley

**Affiliations:** 1 Division of Biomedical Sciences, University of Edinburgh Medical School, George Square, Edinburgh EH8 9XD, UK; 2 Department of Biochemistry, School of Medical Sciences, University of Bristol, Bristol BS8 1TD, UK

## Abstract

**Background:**

Chlamydial bacteria are obligate intracellular pathogens containing a cysteine-rich porin (Major Outer Membrane Protein, MOMP) with important structural and, in many species, immunity-related roles. MOMP forms extensive disulphide bonds with other chlamydial proteins, and is difficult to purify. Leaderless, recombinant MOMPs expressed in *E. coli *have yet to be refolded from inclusion bodies, and although leadered MOMP can be expressed in *E. coli *cells, it often misfolds and aggregates. We aimed to improve the surface expression of correctly folded MOMP to investigate the membrane topology of the protein, and provide a system to display native and modified MOMP epitopes.

**Results:**

*C. trachomatis *MOMP was expressed on the surface of *E. coli *cells (including "porin knockout" cells) after optimizing leader sequence, temperature and medium composition, and the protein was functionally reconstituted at the single-channel level to confirm it was folded correctly. Recombinant MOMP formed oligomers even in the absence of its 9 cysteine residues, and the unmodified protein also formed inter- and intra-subunit disulphide bonds. Its topology was modeled as a (16-stranded) β-barrel, and specific structural predictions were tested by removing each of the four putative surface-exposed loops corresponding to highly immunogenic variable sequence (VS) domains, and one or two of the putative transmembrane strands. The deletion of predicted external loops did not prevent folding and incorporation of MOMP into the *E. coli *outer membrane, in contrast to the removal of predicted transmembrane strands.

**Conclusions:**

*C. trachomatis *MOMP was functionally expressed on the surface of *E. coli *cells under newly optimized conditions. Tests of its predicted membrane topology were consistent with β-barrel oligomers in which major immunogenic regions are displayed on surface-exposed loops. Functional surface expression, coupled with improved understanding of MOMP's topology, could provide modified antigens for immunological studies and vaccination, including live subunit vaccines, and might be useful to co-express MOMP with other chlamydial membrane proteins.

## Background

Every Gram-negative bacterium in the order *Chlamydiales *is an obligate intracellular pathogen [1]. The organisms are dimorphic, and alternate between free-living, infectious "elementary bodies" (EBs) endocytosed by mucosal cells into vesicular inclusions, and metabolically active, intracellular "reticulate bodies" (RBs). RBs replicate and redifferentiate into EBs before being released to infect neighboring cells, and infections (including *Chlamydia muridarum *pneumonitis, an important animal model) are often complicated by a damaging immune response and chronic inflammation. Human genital *C. trachomatis *infections are associated with ectopic pregnancy and infertility, and serovars that target ocular membranes can lead to trachoma and blindness. *Chlamydophila pneumoniae *(*Ch. pneumoniae*) causes pneumonia in the elderly, and colonization of the placenta by *Ch. abortus *causes abortion in ewes (and, occasionally, in women).

Uniquely among bacteria, the chlamydial outer membrane (OM) is reinforced by a network of disulphide bonds [2]. Treatment of EBs with Sarkosyl produces "chlamydial OM complexes" (COMCs) [3] containing three relatively detergent-resistant, cysteine-rich proteins: the Major Outer Membrane Protein (MOMP), encoded by *ompA*, and OmcB and OmcA, encoded by *omp2 *and *omp3*, respectively. MOMP (~40 kDa) is expressed in both EBs and RBs [4]. It contains extensive β-sheet secondary structure and forms large pores [5,6], similar to β-barrel porins found in other outer bacterial membranes (e.g. *E. coli *OmpF). The MOMPs encoded by different *C. trachomatis *serovars share five well-conserved regions and four "variable sequence" (VS) domains [7,8]. *C. trachomatis *VS domains, and homologous regions in MOMPs from other species, could correspond to cysteine-rich surface-exposed loops in a porin β-barrel, and EB MOMP is oxidised and highly cross-linked, making the OM very stable. RBs in contrast are osmotically active with reduced, mainly monomeric, MOMP [9]. MOMP's pore-forming ability is enhanced by reduction [5], compatible with a link between reversible disulphide bond formation and the developmental stage of the bacteria. Supporting this idea, DTT-reduced EBs tend to resemble RBs [5], and native MOMP is monomeric when solubilised in SDS under reducing conditions, but forms monomers, dimers, trimers, tetramers and even larger complexes [e.g. [6,10,11]] under oxidising conditions.

*C. trachomatis *MOMP is highly immunogenic. Antibodies to the protein neutralised EB infectivity [12], and triggered approaches to generate MOMP-based vaccines [e.g. [13,14]]. However, as implied earlier, the immunopathology of chlamydial infections is complicated [15], with T_H1 _type immune responses as well as specific antibodies (T_H2 _responses). MOMP is not equally immunogenic in all *spp*., and it also stimulates T-cell division, including CD4+ and CD8+ T-cells, enhancing IFN-γ secretion [16]. *C. trachomatis *MOMP will probably need to be modified to form a safe and effective subunit vaccine, emphasizing the importance of understanding its structure in more detail.

OmcA and OmcB (the other main components of the COMC) are present as approximately 1 OmcB:2 OmcA:5 MOMP [17]. *Ch. psittaci *(formerly known as *C. psittaci*) OmcA is a 9 kDa lipid-anchored protein with 14 cysteine residues [18], while OmcB (60 kDa) contains 37 cysteines [19]. The Omc proteins may not be integral membrane proteins. Reduced OmcB is water-soluble, and although OmcA remains membrane-associated, it can be readily solubilised when reduced [20], and neither protein was detected on the surface of intact EBs by immunogold labeling [21]. Regardless of their membrane association, OmcB appears to be extensively cross-linked in the periplasm of EBs, forming disulphide bonds with both MOMP and OmcA. Appropriately, both Omc proteins are expressed late in the developmental cycle (from a bicistronic operon), as RBs are reorganized into EBs [22], consistent with the idea that RB MOMP is functional and exchanges nutrients and other factors (possibly including signaling molecules) with the host cell. Extensive disulphide cross-linking in EBs may inactivate the porin, and prevent expansion of the growing bacterial cell wall.

Although MOMP is of major biological and clinical interest, chlamydia only grow in eukaryotic cells, and MOMP is difficult to isolate and purify because it can aggregate when oxidized, or interact with other cysteine-rich chlamydial proteins. As a result, many groups have expressed recombinant MOMP in *E. coli *using full-length *ompA *genes that include the signal sequence to target the translated protein to the OM. Although leadered MOMP can be expressed in a heterologous system [23-25], this approach has proved to be highly problematic, because the protein tends to misfold and aggregate. Koehler *et al*. [26] demonstrated surface-exposure, but with a dramatic reduction in cell viability, including OM disruption and substantial cell lysis (i.e. unincorporated, periplasmic MOMP may have been exposed). Jones *et al*. [27] co-reconstituted recombinant MOMP with endogenous *E. coli *porins, and showed altered solute permeabilities in liposome-swelling assays. Although attributed to novel porin activity, this could have reflected modification of endogenous porins. Wyllie *et al*. [28] pursued an alternative approach with truncated versions of *Ch. abortus *and *Ch. pneumoniae *MOMP, and obtained small amounts of folded proteins without prior denaturation and refolding, sufficient for incorporation into planar bilayers and single-channel recording. Other expression systems, pioneered because of their potential for vaccine delivery, include mammalian COS cells [29] and *Vibrio cholerae *[30].

PorB (37 K), a second putative porin, is also surface-exposed in chlamydia [31]. Recombinant PorB specifically transported dicarboxylates in liposome-swelling assays [32], although. it was used with a C-terminal His tag. The terminal residues of porins normally meet to complete a transmembrane β-strand, and may even be linked by a salt bridge. Being integral to the protein fold, additional terminal residues might affect the conformation and, therefore, the specific function of a porin. We expressed PorB as well as MOMP to help determine the factors affecting chlamydial porin expression, but because of these theoretical concerns concerning porin folding and function, we avoided tagged proteins in the present study, and built on previous work with leadered constructs.

We developed improved conditions for the surface expression of MOMP in *E. coli *cells, and demonstrated unambiguously by single-channel recording that recombinant *C. trachomatis *MOMP folded and formed a functional protein in the absence of many endogenous porins. We showed that MOMP can insert into the outer membrane of *E. coli *cells and form SDS-sensitive oligomers in the absence of cysteine residues, and generated a "working model" of the topology of MOMP to provide structural hypotheses that could be tested by engineering the recombinant protein.

## Results

### Optimised MOMP expression in *E. coli *cells

**Figure 1:**
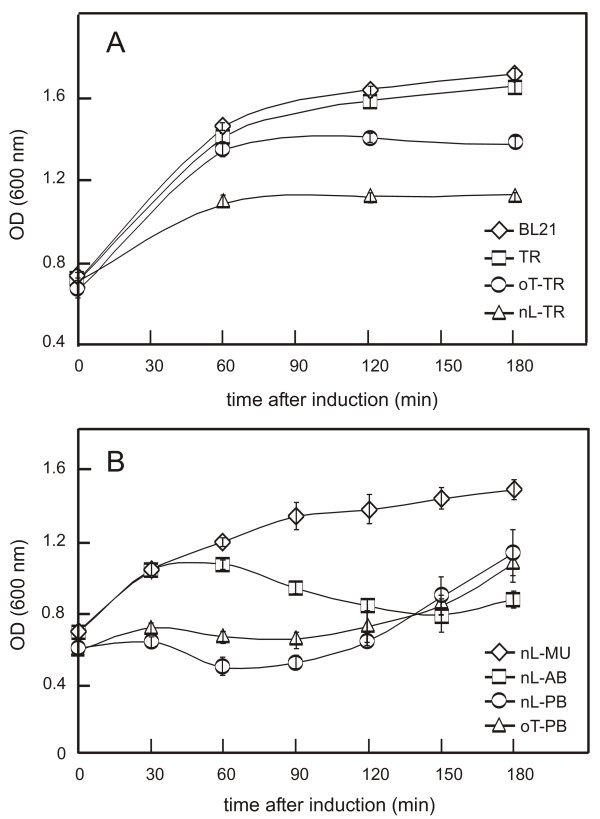
**Effects of leader sequences on the growth of *E. coli *BL21 cells expressing chlamydial porins. **A. Moderate inhibition of bacterial (BL21) growth during expression of *C. trachomatis *MOMP containing native (nL-TR) and OmpT (oT-TR) leaders, contrasting with lack of significant inhibition during expression of mature, leaderless *C. trachomatis *MOMP (TR). B. Slight inhibition of bacterial growth during expression of *C. muridarum *MOMP with its native leader (nL-MU, compare to BL21 in A), contrasting with strong inhibition when *Ch. abortus *MOMP is expressed with its native leader (nL-AB). Cell growth is markedly reduced with both native-leadered PorB (nL-PB) and OmpT-leadered PorB (oT-PB). Note the "recovery" as non-resistant organisms overgrow in β-lactamase-containing cultures (see text). Measurements are means ± SEM (n = 4 independent experiments).

Our first objective was to obtain properly folded recombinant chlamydial porins in the outer membranes of *E. coli *cells. Building on previous work (e.g. [26]), BL21(DE3) cells were transformed with pET-*ompA *or pET-*porB *constructs, and expression was induced by 1 mM IPTG at 37°C after growth to an OD_600 _of 0.6. Compared to the expression of non-leadered proteins (which accumulate in cytoplasmic inclusion bodies), cells expressing leadered porins must transport the immature full-length porin across the inner membrane, cleave the leader sequence in the periplasmic space, and fold and insert the mature protein into the OM. Expression of mature, leaderless *C. trachomatis *MOMP did not inhibit growth compared to non-transformed cells, in contrast to substantial inhibition with full length MOMP (Fig. 1A). To investigate whether different leader sequences could improve processing, *C. trachomatis *MOMP was expressed with the OmpT leader rather than its native leader. Initial growth rates were comparable to those shown by non-transformed cells, and similar to cells expressing mature MOMP (i.e. MOMP without a leader sequence), although the cultures again showed a reduced final cell density.

We next investigated the expression of MOMPs from other chlamydial *spp*. to determine whether the observed effects were specific to *C. trachomatis *MOMP, and we also expressed *C. muridarum *PorB to exclude a universal problem with the expression of all putative chlamydial porins in *E. coli*. The constructs had different effects on cell viability (Fig. 1B). Bacteria expressing *C. muridarum *MOMP grew more slowly than bacteria expressing *C. trachomatis *MOMP, although the bacteria continued to grow slowly throughout the entire period of induction. The growth of bacteria expressing *Ch. abortus *MOMP or *C. muridarum *PorB was markedly reduced, and the density decreased after 30 min. The "recovery" at later stages reflected multiplication of non-expressing cells in the presence of β-lactamase released from dead or dying cells (growth ceased on fresh Ampicillin plates, data not shown). We then changed the leader sequences. The growth of cells expressing *C. trachomatis *MOMP and *C. muridarum *PorB was improved by replacing the native chlamydial leader with the *E. coli *OmpT leader, and the decrease in optical density occurred later in the induction and continued more slowly. In contrast, no significant improvement was seen when *Ch. abortus *MOMP was expressed with the OmpT leader (data not shown).

**Figure 2:**
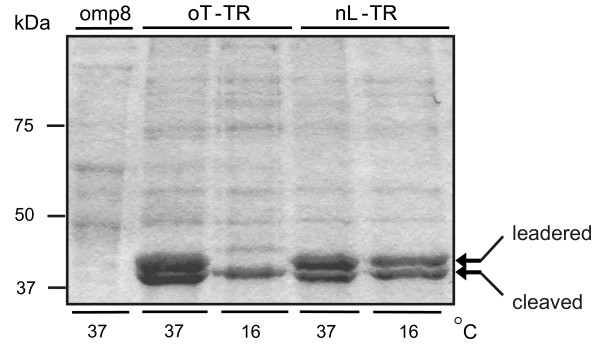
**Expression and processing of *C. trachomatis *MOMP in BL21omp8 cells. **Coomassie-stained 12% (w/v) SDS-PAGE of OM proteins extracted using 1%(w/v) OG (10 μg protein per lane) from cells induced for 4 hrs in LB medium, showing dependence of *C. trachomatis *MOMP expression and processing on temperature and leader sequence. Cells incubated at 16°C and 37°C were induced with 0.1 mM IPTG and 1 mM IPTG, respectively. omp8: non-transformed omp8 cells; oT-TR: cells expressing *C. trachomatis *MOMP with the OmpT leader; nL-TR: cells expressing *C. trachomatis *MOMP with its native leader.

We also expressed full-length constructs in *E. coli *BL21(DE3)omp8 cells lacking expression of the endogenous porins LamB, OmpA, OmpC and OmpF [34]. Toxicity was more pronounced than in unmodified BL21 cells, and after establishing conditions for detergent extraction of recombinant MOMP (Additional Data File #1), expression conditions were further optimised to improve the yield of processed, recombinant protein. Native and OmpT-leadered *C. trachomatis *MOMP constructs were induced rapidly at 37°C with 1 mM IPTG or slowly at 16°C with 0.1 mM IPTG (Fig. 2). At 37°C both versions of MOMP were expressed, and by 4 hours about half the protein was processed, as shown by the doublet band of OM-associated MOMP with and without its signal sequence (Fig. 2). The ~2 kDa difference between the cleaved and non-cleaved protein bands (38 kDa and 40 kDa, respectively), is similar to the difference seen when leadered versions of *E. coli *OmpF are expressed). There was a slight decrease in total protein when MOMP was expressed with its native leader at 16°C, but the proportion of processed protein was unchanged. Although protein decreased following slow induction of MOMP containing the OmpT leader, most of the protein was processed. Based on these observations, slow induction of native-leadered MOMP was carried out in different growth media for prolonged periods. After growing for 6 hours, cultures in LB medium plateaued at an OD_600 _~0.85, after which the cells began to lyse. In contrast, cells cultured in more supportive SOC medium continued to grow steadily, and began to plateau about 12 hours after induction (Additional Data File #2).

### Processing and surface expression of mutagenised and engineered MOMPs

**Figure 3:**
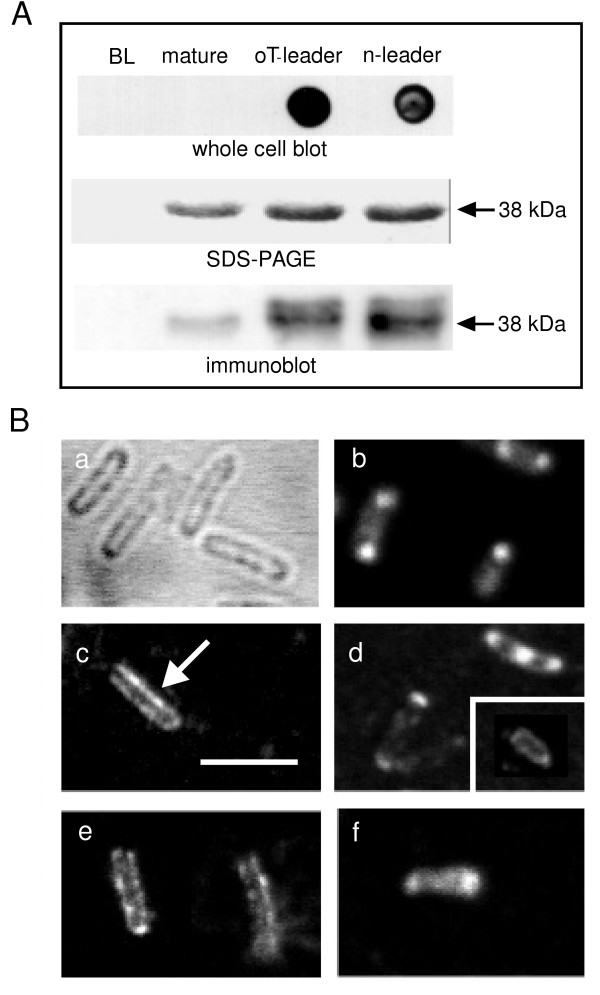
**Insertion of MOMP into the *E. coli *outer membrane. **A. Recombinant *C. trachomatis *MOMP was expressed for 2 hrs at 25°C from constructs encoding either no leader (mature), the OmpT (oT) leader, or the native (n) leader, and immunodetected on the surface of intact BL21 cells (upper panel) using a specific anti-MOMP polyclonal Ab. Non-expressing BL21 cells (BL) show no signal, and mature, leaderless MOMP does not reach the cell surface. The middle and lower panels show, respectively, SDS-PAGE and immunoblot analyses of the corresponding recombinant proteins under these conditions (prepared as in Fig. 2). Note the presence of some unprocessed protein, revealed by the immunoblots of leadered protein expression. Representative of 3 similar experiments. B. Immunofluorescence confocal microscopy (panels b–f), with examples of unstained cells (panel a); cells permeabilised and stained after expressing mature, non-leadered MOMP (panel b); cells expressing OmpT-leadered MOMP stained before (panel c) and after permeabilisation (panel d, with inset permeabilised omp8 cell after 12 hrs induction at 16°C); cells expressing native-leadered MOMP under corresponding conditions (panels e and f, respectively). The scale bar (panel c) is 3 μm, and the arrow points out membrane staining. Representative of 3 similar experiments.

Given the known difficulties associated with protein misfolding and aggregation (e.g. [23-26]), a particular problem for chlamydial MOMPs compared to other bacterial porins, our next objective was to determine whether MOMP was actually inserted into the *E. coli *outer membrane. Although recombinant MOMP was associated with the OM fraction following subcellular fractionation, the observation that its leader sequence was not always cleaved (Fig. 2) suggested that some leadered protein co-fractionated with OMs, possibly as a peripheral membrane protein. This raised the possibility that even cleaved recombinant proteins might not be fully integrated into the OM. To determine whether processed MOMP was actually inserted into (and across) the OM, we carried out whole cell immunoblots to probe for the presence of MOMP epitopes on the surface of intact *E. coli *BL21 cells. Because of the importance of reduced temperature (Fig. 2), we carried out inductions for whole cell immunoblotting at 37°C, 16°C and an intermediate temperature of 25°C. MOMP was incorporated into the OM at both 25°C and 16°C, when induced in the presence of either 1 mM or 0.1 mM IPTG, respectively. Expression and processing were more rapid at 25°C, and because the presence of some unprocessed protein was irrelevant in this experiment, we induced the cells at 25°C for 2 hrs. Non-transformed BL21 cells, or cells transformed with an empty plasmid, and BL21 cells transformed with constructs encoding mature, leaderless *C. trachomatis *MOMP, or with OmpT-leadered MOMP and native leadered-MOMP, were applied to a nitrocellulose membrane (avoiding methanol-activated PVDF, and the risk of OM permeabilisation and exposure of periplasmic MOMP), and probed with anti-MOMP pAb (Fig. 3A).

The absence of a signal from control cells and cells expressing MOMP in its non-leadered, mature form confirmed the incubation and blotting conditions did not cause cell lysis and expose unincorporated protein. Both OmpT- and native-leadered MOMP were detected on the cell surface (Fig. 3A, whole cell blots), confirming they were inserted into the OM. Unfortunately, BL21omp8 cells were too fragile to survive the same blotting procedure. SDS-PAGE analysis of OG-solubilised OM fractions (Fig. 3A, middle panel) confirmed MOMP expression and processing, although parallel immunoblots (Fig. 3A, lower panel) showed faint additional bands of ~40 kDa for the leadered proteins, indicating that processing was incomplete, as expected. Parallel immunofluorescence data (Fig. 3B) showed MOMP was confined to cytoplasmic inclusion bodies containing the mature protein when the appropriate cells were fixed and permeabilised before staining (Fig. 3B, panel b). As expected, staining was absent when the antibody was applied before permeabilisation (data not shown). However, OM staining was seen for MOMP expressed with both the OmpT leader and the native leader (panels c and e, respectively). When these cells were permeabilised before staining (panels d and f, respectively), immunoreactive protein was also noted internally, as expected (e.g. Fig. 3A, lower panel), although reduced or absent in BL21omp8 cells induced for 12 hrs at 16°C in more supportive SOC medium (Fig. 3B, inset in panel d).

We concluded that MOMP constructs encoding appropriate leaders could be expressed in *E. coli*, cross the inner membrane, and be processed in the periplasm. Furthermore, under modified incubation and induction conditions (especially at reduced temperatures, and in the relatively supportive medium SOC), MOMP could be folded and incorporated into the outer membrane.

### Membrane topology of MOMP

**Figure 4:**
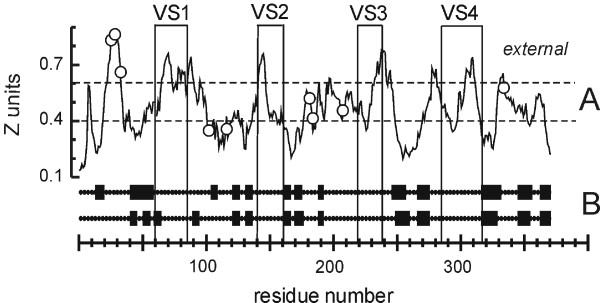
**Membrane topology and secondary structure predictions for *C. trachomatis *MOMP. **A. "Membrane crossing" prediction. Surface-exposed VS domains and cysteine residues are indicated by boxes and circles, respectively. A "complete membrane crossing" corresponds to a (contiguous) region of the plot that crosses both dotted lines in sequence, where the dotted lines represent the internal (periplasmic) and external borders of the outer membrane (in Z units). B. Two independent β-strand predictions, TMBETA [38] (upper line) and B2TMPRED [37] (below). The strands are boxed. The residue numbers refer to the mature (processed) protein.

Having confirmed that *C. trachomatis *MOMP was inserted into the OM of *E. coli *cells, we set out to investigate how the protein was organized in the membrane. While noting that predictive algorithms must always be deployed with care, and with reference to established findings for a given protein, we first analyzed MOMP's primary sequence for membrane crossings using a neural network trained with OM proteins of known structure [36]. The analysis (Fig. 4A) showed 16 membrane crossings. As expected, the VS domains of *C. trachomatis *MOMP generally corresponded to regions of the protein predicted to be extracellular.

**Figure 5:**
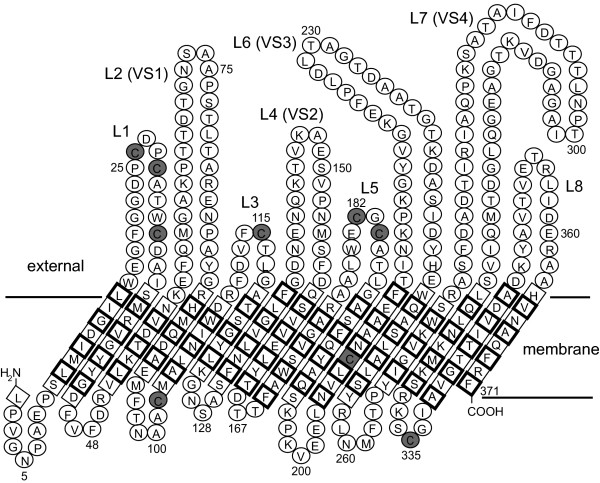
**Model of *C. trachomatis *MOMP. **The MOMP β-barrel is broken apart at the putative N-C salt bridge in β-strand 16 to display the protein in a 2-D projection. Residues in the transmembrane strands are boxed, with a bold border to indicate side chains facing the bilayer. External loops (including constituent VS domains) are labeled, and cysteine residues are shaded.

We then reanalyzed the sequence using two β-strand prediction programs (Fig. 4B). The combined analysis revealed a total of 16 strands, corresponding numerically to the initial "membrane crossing" prediction (which does not on its own appear to be sufficient to identify the specific extramembrane domains). We discarded the strand coinciding with VS1 in B2TMPRED (see Methods) because VS domains are likely to be extracellular loops, and inserted an extra strand between G210 and S218 to bring the chain back across the membrane, so that all 4 VS domains remained external. Minor adjustments were made to accommodate known constraints on β-strand organization and porin structures [39,40]. The final working model (Fig. 5) provided testable hypotheses concerning the pattern of transmembrane folding. All the cysteine residues were predicted to be accessible for inter- or intrasubunit disulphide bond formation or cross-linking with other proteins. Most were predicted to be external, but two were periplasmic. Although one thiol group was in a predicted transmembrane domain, it faced the central water-filled pore rather than the lipid bilayer, where it could potentially interact with a cysteine thiol on a pore-confined loop.

**Figure 6:**
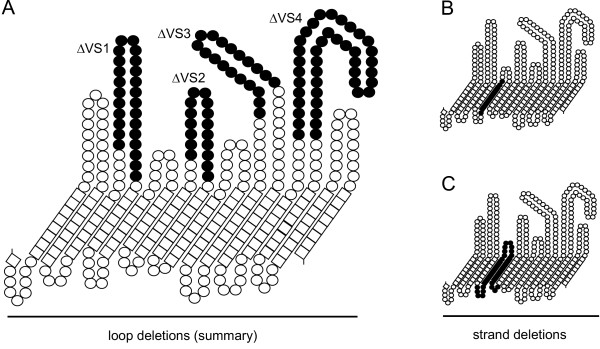
**Loop and strand deletion maps. **A. Summary of individual loop deletions (filled residues) providing four *C. trachomatis *MOMP proteins deficient in VS domains 1, 2, 3 or 4 (designated ΔVS1-4, respectively). B. Deletion of predicted β-strand 5 and its associated internal loop, designated in the text as Δβ5. C Deletion of predicted β-strands 5 and 6 and their associated internal and external loops, designated in the text as Δβ5,6.

We designed four *C. trachomatis *MOMP constructs (with intact cysteines and native leaders, to correspond exactly in these respects to the "wild-type" protein) in which substantial regions of VS domains 1, 2, 3 or 4 (shown in Fig. 6A) were deleted, to test the prediction that these domains are surface-exposed loops that can be shortened without compromising the main β-barrel fold and membrane insertion. The region removed from VS1 was G63 to Y87; from VS2, E141 to F156; from VS3, Y220 to G238; and from VS4, D278 to T318. Our strategy (see Methods) resulted in some mutations. Most were conservative changes (M62T in VS1, T239V in VS3 and A277V in VS4), apart from G219D in VS3. However, our topology prediction placed this residue in an external loop, where the additional charge was unlikely to be significant. We also generated another pair of constructs with deletions of either one or two of the predicted β-strands between VS domain 1 and VS domain 2 (summarized in Fig. 6B–C), in an attempt to disrupt the formation of OM-inserting β-barrels. These constructs were designated: Δβ5, with removal of E95 to F111 (with no residue changes) and Δβ5,6, with removal of F97 to A129 (with 2 changes, E95D and M96V).

**Figure 7:**
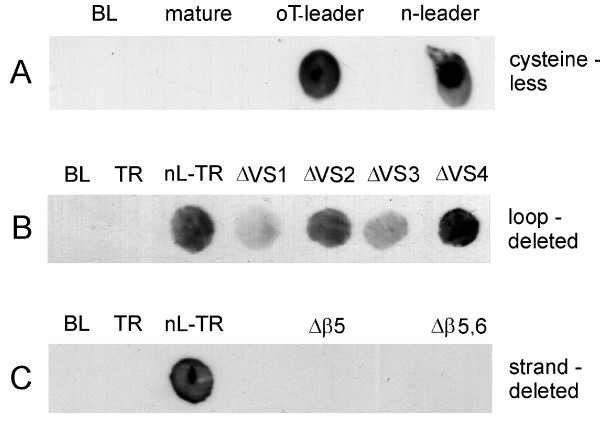
**Effects of cysteine mutagenesis and loop and strand deletions on surface exposure of MOMP. **A. Replacement of all 9 cysteine residues in *C. trachomatis *MOMP by alanine residues fails to prevent surface exposure. BL: non-expressing BL21 cells; mature: cells expressing mature MOMP without a leader sequence; oT-leader and n-leader, cells expressing MOMPs with ompT and native leaders, respectively. B. Removal of loops containing putative external VS domains (shown in Fig. 6A) fails to prevent surface exposure. TR: cells expressing mature MOMP; nL-TR: unmodified MOMP with its native leader. C. Removal of putative β-strand regions illustrated in Fig. 6B–C prevents surface exposure. Each result is representative of at least 3 similar experiments.

Before expressing the cDNAs encoding putative loop or strand deletions, we re-examined the expression and OM insertion of full-length *C. trachomatis *MOMP using a construct in which all 9 cysteine residues (Fig. 4A, circles; Fig. 5, shaded residues) were replaced by alanine. The results (Fig. 7A) were similar to those for the non-mutagenised protein, showing that folding and membrane insertion could proceed without cysteine residues and without the controlled formation of disulphide bonds (as it may do in RBs). We then expressed each of the "loop-deleted" MOMP proteins in BL21 cells. All four were detected on the cell surface (Fig. 7B), demonstrating incorporation into the OM. In contrast, recombinant proteins expressed from constructs with putative β-stand deletions were not detectable on the surface of *E. coli *cells (Fig. 7). We considered the unusual possibility that all the epitopes in the "strand-deleted" proteins might have been unreactive in the *E. coli *membrane, due to masking or oligomerisation, but suspension of the cells in Tris (rather than phosphate) buffer (100 mM NaCl, 50 mM Tris-HCl, pH 7.4), or the addition of 2 mM EDTA, failed to "unmask" any immunoreactivity (Additional Data File #3).

### MOMP forms oligomers in the *E. coli *outer membrane

Native MOMPs are difficult to purify free from other chlamydial proteins [6], precluding firm conclusions about native subunit structure, especially in the absence of protein (cysteine) oxidation. In preliminary investigations of the subunit organisation of recombinant MOMP, we noted that the recombinant protein did not form SDS-resistant oligomers (Additional Data File #4). However, unlike trimeric *E. coli *porins [40], oligomers of isolated MOMP, away from their normal membrane environment [6], may be unstable in the presence of SDS, so we subjected detergent-solubilised OM extracts to large-scale non-denaturing GE chromatography in milder detergents. For these and all subsequent experiments, MOMP was expressed in BL21omp8 cells with the OmpT leader (in SOC medium, at 16°C), to exclude heterooligomers containing endogenous *E. coli *porins, and minimize uninserted periplasmic protein, respectively.

**Figure 8:**
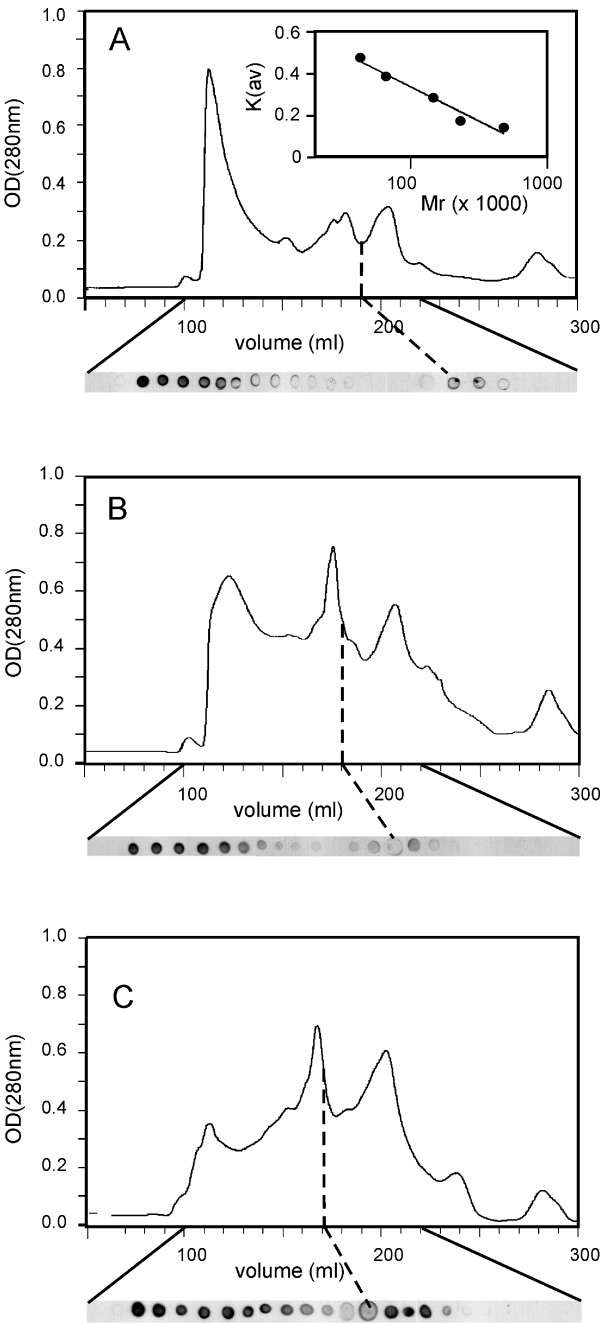
**Gel-exclusion chromatography of solubilised MOMPs. **A. GE analysis of *C. trachomatis *MOMP expressed in BL21omp8 cells with the OmpT leader, solubilised from an OM preparation in 1% (w/v) LDAO. Immuno-dotblots from successive 5 ml fractions between 100–220 ml (shown below the absorbance trace) reveal the appearance of high-molecular mass MOMP aggregates after the void volume, with a second immunoreactive peak at 195 ml (range 185–205 ml), corresponding to a molecular mass of 80 kDa (note inset column calibration trace, also in the presence of detergent). B. GE analysis of *C. trachomatis *MOMP expressed as in A but solubilised in 1% (w/v) Zwittergent 3–14. Oligomeric MOMP peaks at 180 ml (range 170–190 ml), corresponding to a molecular mass of 120 kDa. C. GE analysis of *C. trachomatis *MOMP in which all 9 cysteines were changed to alanine, expressed and solubilised as in B. Oligomeric MOMP peaks at 170 ml (range 160–190 ml), corresponding to a molecular mass of 160 kDa. Each trace is representative of at least 3 experiments.

We carried out GE chromatography in LDAO or Zwittergent 3–14 (having previously noted these to be cheaper but equally effective detergents to replace OG, Additional Data File #1), with excess (5 mM) DTT in the presence of MOMP cysteine residues (calibrating the column in the presence of detergent). Under these conditions, MOMP appeared to form oligomers containing 2–4 subunits, although some recombinant MOMP always formed higher-order oligomers (Fig. 8). Similar results were obtained after repeating each experiment at least twice. The apparent subunit stoichiometry of recombinant MOMP depended on the detergent, with putative dimers in LDAO, and trimers or tetramers in Zwittergent 3–14, depending on the presence or absence of cysteine residues, respectively. However, it should be emphasized that only the major quaternary species was identified in each case. The presence of oligomers in LDAO or Zwittergent 3–14 contrasted with the absence of SDS-resistant oligomers during SDS-PAGE, and oligomer formation even in the absence of cysteine residues argued against an essential role for disulphide bonds.

**Figure 9:**
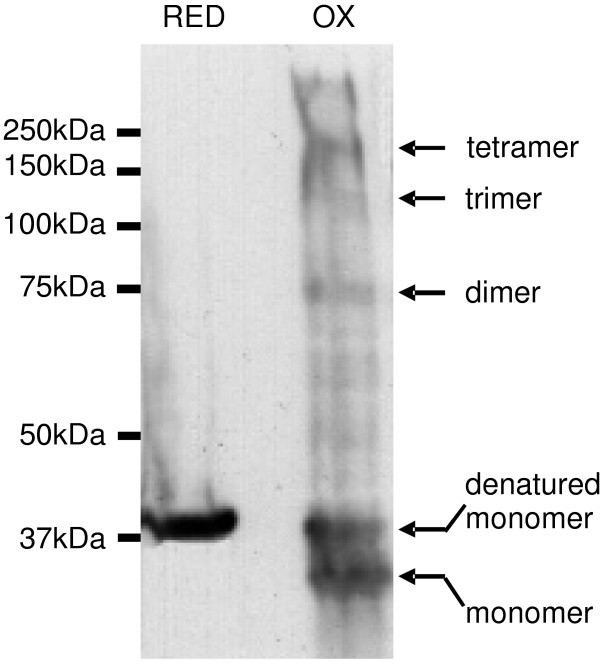
**Cysteine cross-linking of recombinant *C. trachomatis *MOMP. **MOMP expressed in BL21omp8 cells with the OmpT leader was covalently cross-linked as described in the text and OM proteins were separated by reducing (RED) and non-reducing (OX) SDS-PAGE, followed by Western blotting using a specific anti-MOMP antibody. The positions of molecular mass markers are shown to the left, and the proposed identities of bands are shown to the right.

We also investigated the subunit organization of MOMP by covalent cross-linking following expression and insertion into BL21omp8 OMs, by removing DTT to allow *in situ *cysteine oxidation by dissolved oxygen. OM proteins were then incubated in SDS sample buffer with or without reducing agent at room temperature for 10 mins, separated by SDS-PAGE, and detected by Western blotting (Fig. 9). Reduced MOMP appeared as a single band of ~38 kDa, but non-reduced MOMP occupied several distinct bands. SDS-denatured, monomeric MOMP appeared as a band of ~38 kDa (labeled "denatured monomer"), corresponding to the reduced sample. However, monomeric MOMP also formed a band of ~35 kDa, running "ahead" of its normal apparent molecular mass, as previously seen with "folded" porin monomers [41,42]. Additional, fainter bands at higher molecular masses corresponded to dimers, tetramers and possible trimers (~80 kDa, ~160 kDa and ~120 kDa, respectively), similar to the findings following GE chromatography, with an upper band of aggregated protein that failed to enter the gel.

### Surface-expressed MOMP is functional

**Figure 10:**
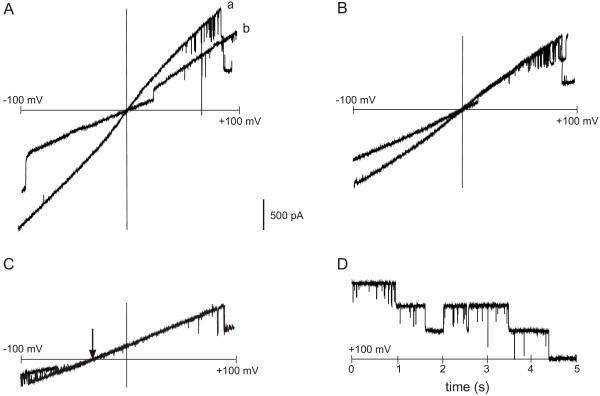
**Single-channel analysis of recombinant *C. trachomatis *MOMP from BL21omp8 cells. **A. Channel currents in response to two transmembrane voltage ramps (-100 mV to +100 mV), with 1 M KCl *cis *and *trans*. Up to 3 channels are open during ramp (a), and 2 of the channels close in succession at about +80 mV. In ramp (b), 1 channel is open at 0 mV, and 2 channels are open at +100 mV. B. Channel currents in the presence of 500 mM KCl *cis *and *trans *during two voltage ramps (as in A). Note the reduction in single-current amplitudes, and current reversal at 0 mV as in A. C. Currents during two voltage ramps in asymmetric KCl, 500 mM *cis vs *50 mM *trans*. Note the shift in equilibrium potential (E_r_) from 0 mV to -30 mV (arrowed). Positive (upgoing) currents at 0 mV indicate a net flux of K^+ ^*cis *to *trans *in the absence of an electrical driving force. **D. **Equilibrium currents in symmetric 500 mM KCl at a constant holding potential of +100 mV, showing brief single-channel closures and voltage-dependent inactivation of all 3 channels within 5 s.

Fully processed and correctly folded MOMP should function as a porin-like ion channel [6]. We tested this crucial prediction by expressing "wild-type" full-length recombinant *C. trachomatis *MOMP in BL21omp8 cells which express only a small subset of native *E. coli *porins, and not OmpF or OmpC [34]. We then functionally reconstituted solubilised BL21omp8 OM protein GE fractions in voltage-clamped planar lipid bilayers. Fractions containing "oligomeric" MOMP complexes gave rise to large-conductance, porin-like ion channels (Fig. 10). Similar channels were recorded irrespective of whether the detergent was LDAO or Zwittergent 3–14 (using fractions corresponding to 195 ml or 180 ml, respectively). The channels were voltage-dependent, closing at relatively high holding potentials (e.g. + or - 100 mV), but remaining open around 0 mV. The single-channel conductance in symmetric 500 mM KCl was 480 ± 19 pS (mean ± SEM, n = 6 independent experiments), and the reversal potential in 500 mM *vs *50 mM KCl (*cis vs trans*) was -31 ± 1.5 mV (mean ± SEM, n = 9 independent experiments). This corresponded to a relative cation *vs *anion selectivity of 3.8 under these specific ionic conditions. Control preparations (detailed under Methods), including membrane proteins from control BL21omp8 cells subjected to the same experimental conditions, where OM proteins were solubilised and subjected to GE chromatography in exactly the same way, did not give rise to similar channel activity (6 experiments).

## Discussion

Functional reconstitution of recombinant *C. trachomatis *MOMP at the single-channel (single molecule) level from cells lacking many endogenous porins provides very strong evidence that MOMP adopted its native fold when expressed *in E. coli *under suitable conditions. Although a leadered version of recombinant chlamydial MOMP was expressed and functionally analysed previously [27], membranes containing the protein were co-reconstituted with endogenous *E. coli *porins for liposome-swelling studies. Although MOMP may have contributed additional porin-like activity, functional modification of endogenous porins could not be ruled out.

Interestingly, the successful expression and processing of recombinant chlamydial porins in *E. coli *cells depends on the precise leader sequence, as well as on the specific protein. PorB is less "toxic" with its native leader, in contrast to MOMP, which is less "toxic" with the *E. coli *OmpT leader, and native-leadered *C. muridarum *MOMP is less deleterious to *E. coli *than *Ch. abortus *MOMP. Although a full investigation of the role of leader sequences could not be undertaken here, it is known that successful OM insertion, as well as prior transport across the inner membrane and processing, is also signal sequence-dependent. For example, a large proportion of *E. coli *LamB porins with signal sequence mutations remained "tethered" to the inner membrane (probably by their unprocessed signal sequence), even though the protein was also closely associated with the OM [43]. For *C. trachomatis *MOMP, use of the Omp-T leader and induction at 16°C (not induction at 37°C, as previously employed), in either "wild-type" cells or "porin knockout" cells in a supportive medium (SOC), provides improved processing and OM insertion, and there is also significant insertion at 25°C in "wild-type" *E. coli*.

The single-channel properties of *C. trachomatis *MOMP are consistent with previous data on bacterial [40] and putative chlamydial [6] porins. In particular, the channels show "bell-shaped" voltage-dependent gating and are mainly open around ~0 mV, with very high conductances (close to the saturating conductances predicted for a large water-filled pore) and poor ionic selectivity, showing only a slight preference (~4:1) for cations over anions (using a Nernst-Plank analysis because relatively wide, water-filled porin channels are probably electroneutral [35], and poorly-described by electrodiffusion theory). The channels often appeared in groups of three, as might be expected for a trimeric "triple-barrelled" porin (e.g. Fig. 10). However, unless the channels were randomly incorporated into the bilayer (which is difficult to demonstrate), these complexes may represent a selected sub-population.

Despite the lack of sequence similarity to known bacterial porins, a combination of different predictive approaches (none of which was entirely satisfactory in isolation), set in the context of elegant and pioneering work from many laboratories on the properties of VS domains, predicted that *C. trachomatis *MOMP, like putative porins in the intracellular pathogens *Burkholderia thailandensis *and *B. pseudomallei *[44], could be a 16-stranded β-barrel. Our working model pays due attention to the construction principles for β-barrels [39,40]. The N and C termini complete final strand 16, the periplasmic turns are short, and most external loops are long and include the immunogenic VS domains. The barrel surface in contact with the bilayer consists largely (though not exclusively) of hydrophobic side chains, and all 18 strand residues with charged side chains project into the pore to line the central water-filled central channel. 6 cysteines lie in extracellular loops, and 2 periplasmic cysteines lie on opposite sides of the barrel where they are unlikely to form an intrasubunit disulphide bond, although they could form intersubunit bonds, or bonds with other proteins. A single membrane thiol projects into the barrel pore, where it could be involved in disulphide bond formation if a loop (e.g. L1) were to fold into the barrel.

Our working model for the membrane topology of *C. trachomatis *MOMP differs in some significant respects from the recent prediction for *C. muridarum *MOMP [45] (which was based partly on hydrophobicity plots). Although both studies predict that MOMPs are 16-stranded β-barrels with an average strand length of ~8 residues, periplasmic thiols are absent from the *C. muridarum *prediction. This would preclude the significant interactions with OmcB and OmcA, described in the Background. We also assigned L2, 4, 6 and 7 as *C. trachomatis *VS domains, not L2, 3, 5 and 6, the homologous regions in *C. muridarum *MOMP. Experimental tests of the predicted membrane topology of *C. trachomatis *MOMP are consistent with our model, because individual VS domains can be substantially truncated without preventing incorporation of the protein into the bacterial OM. If MOMP is a β-barrel porin, as suggested, and VS domains are confined to specific extracellular loops, it is conceivable that MOMP can continue to fold into a β-barrel in the absence of one of these domains. On the other hand, the removal of β-strands would disrupt folding. Removal of a single strand, bringing periplasmic residues into direct contact with external residues, is predicted to be particularly destructive to the global fold. Removal of more than one strand might be better tolerated, provided the β-barrel can form with a significantly reduced diameter. In practice, it appears that *C. trachomatis *MOMP cannot accommodate either type of strand modification.

GE chromatography suggested that MOMP forms oligomers in the presence of Zwittergent or LDAO, and in line with these findings, *in situ *cysteine cross-linking of recombinant MOMP in *E. coli *OMs revealed oligomeric MOMP complexes, together with a species of folded or partially-folded MOMP monomers containing at least one intramolecular disulphide bond. This species contrasts with reduced, denatured MOMP monomers seen when chlamydial MOMP is solubilised directly from OMs (or native EBs [6]). However, the exact stoichiometry of MOMP oligomers in the *E. coli *OM remains uncertain because our size estimates for the oligomers, and thus their stoichiometries, may be too high because of uncorrected bound detergent. Also, it is clear that the stability of MOMP oligomers is detergent-dependent.

Native *Ch. abortus *MOMP forms SDS-resistant oligomers of ~100 K [6], unlike the SDS-unstable MOMP oligomers isolated from *E. coli *OMs. We speculate that this may be because native MOMP oligomers are stabilised by interactions with other chlamydial components (e.g. co-purified Omp90 [6]), and possibly also by disulphide bonds. Disulphide bond formation (whether transient or permanent) does not appear to be essential during protein folding and OM insertion, because a cysteine-free mutant can be fully processed (Fig. 7A) and can also form oligomers. Overall, our results show that the subunit stoichiometry of detergent-solubilised MOMPs expressed and processed in *E. coli *is detergent-dependent, that MOMP subunits can be cross-linked by disulphide bridges, and that folded monomers contain at least one intrasubunit disulphide bond (Fig. 8).

## Conclusions

*C. trachomatis *MOMP, an immunodominant, cysteine-rich, chlamydial surface protein of crucial importance in the immune response to infection, is a major subunit vaccine target. However, unlike many other bacterial porins, it has been difficult to refold from inclusion bodies or to achieve and demonstrate functional surface expression. This study is the first to report unambiguous functional analysis, by single-channel recording, of recombinant chlamydial MOMP recovered from bacterial outer membranes. The modified expression system described in the present study provided a means to test specific hypotheses provided by a working model for the *C. trachomatis *protein. However, although our results are consistent with a working model of MOMP as a 16-stranded β-barrel, more mutations or other approaches are needed before a specific model can be accepted. The protein also formed oligomers, even in the complete absence of cysteine residues. The surface display of modified, functional MOMP in *E. coli *cells (potential vehicles for a live, subunit vaccine), together with a working topological model, could guide the removal of unwanted or harmful epitopes from engineered proteins, and it might also be possible to display external loops containing specific MOMP epitopes on other porin "scaffolds" in living cells. However, it is important to note that such approaches will be limited if essential disulphide bonds in the native chlamydial envelope, including bonds involving non-MOMP cysteines, stabilise the conformation of key immunogenic VS domains.

## Methods

### DNA manipulations

*C. trachomatis ompA *(corresponding to X62918, from the Da serovar) and *Ch. abortus ompA *were cloned without their leader sequences into the *Nde*-I/*Nco*-I sites of pET22b(+) (Novagen) after destroying an internal *Nde*-I site in *C. trachomatis ompA *by Quik-Change PCR mutagenesis (Stratagene). This did not alter the encoded protein. *C. muridarum ompA *and *porB *were amplified with and without their leaders from genomic DNA and cloned into the *Nde*-I/*Bam-*HI sites and *Nde*-I/*Nco*-I sites, respectively, of the same vector (*C. muridarum ompA *also required null mutation removal of an internal *Nde*-I site). The *E. coli *OmpT protease leader sequence or the native *C. trachomatis *MOMP leader sequence was added to the 5' end of the leaderless *C. trachomatis *and *Ch. abortus *inserts by sequential gene extension PCR using three overlapping primers. A 5' *Nde*-I site was again used to provide the starting methionine codon in the final full-length construct. Quik-Change PCR was also used to create pairs of unique internal restriction sites in native-leadered *C. trachomatis ompA *to permit the deletion of specific domains by plasmid restriction and religation [33]. These sites were: for VS1, *Age*-I; for VS2, *Bcl*-I; and for VS3, VS4 and the predicted β-strands, *Aat-*II. Successful deletions were confirmed by hemi-nested single-colony PCR (using *Taq *polymerase) to identify clones that could be amplified by gene-spanning primers but not by primers complementary to regions that had been removed. We also generated *C. trachomatis *MOMP expression constructs containing inserts in which all 9 cysteine residues (C26, C29, C33, C102, C115, C182, C184, C207 and C335) were replaced by alanine using Quik-Change PCR. Most of the modifications were carried out in a pSTBlue-I/NovaBlue system, and the fidelity of each insert was confirmed by automated DNA sequencing (MWG Biotech).

### Protein expression and recovery

*E. coli *BL21(DE3) or BL21(DE3)omp8 [34] cells were harvested from cultures of LB (Luria-Bertani) medium (10 g/l Bacto tryptone, 5 g/l yeast extract, 10 g/l NaCl, pH 7.0) or SOC medium (20 g/l Bacto tryptone, 5 g/l yeast extract, 0.5 g/l NaCl, 20 mM glucose, pH 7.0) by centrifugation at 6,000 × g for 5 mins after inductions as described in the Results section, and washed in 50 ml phosphate buffered saline (PBS). The cell pellet was resuspended in 5 ml TEN buffer (50 mM Tris-HCl, pH 8.0, 10 mM EDTA, 100 mM NaCl) containing 1 mg lysozyme and incubated for 30 min. at room temperature. Following sonication (6 × 15 s, 6 μm amplitude, Sanyo Soniprep 150 sonicator) the cell lysate was incubated with 20 U/ml Benzonase (Novagen) for 15 min at room temperature. OM fragments were pelleted by centrifugation at 15,000 × g for 10 min, and washed twice in 20 ml TEN buffer. Membrane proteins were solubilised by resuspending the pellet in 6 ml solubilisation buffer containing 50 mM Tris-HCl, pH 8.0, 1 mM EDTA, 50 mM NaCl and 10 mM DTT with either 1% (w/v) octyl glucoside (OG, Anatrace), 1% (w/v) lauryl (dodecyl) dimethylamine oxide (L(D)DAO, Anatrace) or 1% (w/v) Zwittergent 3–14 (Anzergent 3–14, Anatrace), and incubating at 37°C for 1 hour. The solution was clarified by ultracentrifugation (Beckman TLA-100) for 20 mins at 100,000 rpm. Protein concentrations were determined after TCA precipitation.

### SDS-PAGE and Western blotting

Unless otherwise indicated, SDS-PAGE was carried out under reducing conditions using 10–12% (w/v) gels. Molecular masses were estimated from plots of relative mobility *vs *the logarithm of the molecular mass of Precision Plus unstained protein markers (BioRad). For Western blotting, proteins were electrophoretically transferred to PVDF membranes under conditions compatible with the transfer of high-MW proteins including native MOMP oligomers [6]. The membranes were blocked in 5% (w/v) non-fat milk in PBS-T (0.005% (v/v) Tween-20 in PBS) then incubated in 1:5000 goat anti-*C. trachomatis *MOMP antibody (Fitzgerald International) for 1 hour at room temperature. Following 2 × 30 sec and 3 × 5 min washes in PBS-T, membranes were incubated in 1:10,000 HRP-conjugated anti-goat/sheep antibody (Sigma) for 1 hr at room temperature. After washing, immunoreactive proteins were detected by ECL.

### Whole cell immunoblotting and immunofluorescence

10 ml of LB medium was seeded 1:100 with cultures grown to saturation overnight, and incubated until the OD reached 0.6. The cells were pelleted by centrifugation (6,000 g × 10 mins) and resuspended in fresh medium. Following incubation at the selected temperature for 10 mins, 0.1–1 mM IPTG was added and incubation was continued for another 2–16 hrs. Intact cells were harvested by gentle centrifugation (4,500 g × 5 mins) and washed in 1 ml PBS. The pellets were resuspended in 200 μl PBS, and 10 μl was applied to a nitrocellulose membrane and allowed to dry. The membrane was blocked and probed with anti-*C. trachomatis *MOMP polyclonal antibody as described above. Immunofluorescence was carried out as described previously [26], with fixation and permeabilisation either before or after immunostaining, using 1:200 dilutions of the above primary antibody and fluorescein-conjugated anti-goat secondary antibody (Sigma). The cells were then observed by bright field, phase contrast and fluorescence microscopy using a Leica TCS-NT confocal microscope.

### Gel-exclusion chromatography

Solublised OM proteins were separated by GE chromatography using a high resolution 26/60 HiLoad Superdex 200 prep grade column (Amersham Pharmacia Biotech) freshly equilibrated in 50 mM Tris-HCl (pH 8.0), 1 mM EDTA, 50 mM NaCl and 5 mM DTT (omitting the latter for the cysteine-less MOMP mutant). The buffer also contained either 0.05% (w/v) LDAO or 0.05% (w/v) Zwittergent 3–14. 2 ml aliquots of solubilised OM proteins (containing up to 10 mg protein, solubilised as described earlier under protein expression) were loaded, and the column was eluted with the same buffer for 800 min. at a flow rate of 0.5 ml/min. 5 ml fractions were collected and 10 μl of each protein-containing fraction was deposited onto a pre-prepared PVDF membrane and probed for MOMP as described earlier under Western blotting. The column (*V*_*t *_320 ml) was calibrated in the presence of detergent using standard proteins. *V*_*0 *_(the void volume) was 115 ml, and *K*_*av *_was calculated as (*V*_*e *_- *V*_*0*_)/(*V*_*t *_- *V*_*0*_), where *V*_*e *_is the elution volume.

### Bilayer reconstitution and single-channel analysis

Planar bilayers were cast from diphytanoyl phosphatidylcholine (Avanti) between two 0.5 ml chambers containing 50 mM KCl, 20 mM Tris-HCl (pH 8.0) and 1 mM DTT, designated *cis *and *trans *[6]. The *cis *chamber was voltage clamped with respect to the *trans *chamber using an Axon 200B amplifier or a Biologic RK300 amplifier. 1–5 μl aliquots of pre-diluted solubilised proteins (containing up to 10 ng protein and no more than 5 ng detergent) were added to the *cis *chamber, followed by aliquots of 5 M KCl to raise the salt concentration to 500 mM. Channel incorporation usually occurred within 30 min, accelerated by switching the holding potential between +/- 60 mV. Experimental protocols were programmed and the digitised data were low-pass filtered (1 kHz, 8-pole Bessel-type response) and recorded using pClamp8 software (Axon Instruments), and analysed offline. The bilayer potential was slowly and repeatedly ramped between -100 mV and +100 mV (each sweep taking 32 s) in the presence of an asymmetric (500 mM *vs *50 mM, *cis vs trans*) gradient of KCl, or with equimolar 500 mM or 1 M KCl. At least 3 voltage ramps were recorded and analysed for each experiment, and equilibrium recordings were obtained at defined holding potentials. Holding potentials refer to the *cis *chamber, and upgoing deflections represent net movement of cations from *cis *to *trans *or of anions from *trans *to *cis*. Relative ionic permeabilities were determined from the equilibrium solution of the Nernst-Planck flux equations [35]. When the cation and anion fluxes are equal:


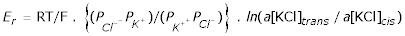


*E*_*r *_is the equilibrium (zero current, or reversal) potential in asymmetric KCl, and R, T and F have their usual significance. 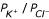 (the permeability ratio of K^+ ^to Cl^-^) was calculated using appropriate activity coefficients (*a*) from standard tables. Control experiments were carried out using equivalent amounts of detergent and with equivalent amounts of solubilised OM proteins purified from non-transformed bacteria, selecting identical GE column fractions.

### Membrane topology prediction

The number of membrane crossings was predicted using a neural network based-outer membrane protein topology prediction program trained with known porins [36]. We discounted a predicted membrane crossing very near the N-terminus that was only apparent after numerical rounding. β-strands were predicted independently by similar computational approaches using B2TMPRED [37] and TMBETA [38], respectively. The three predictions were combined and adjusted manually, taking account of the accessibility of the VS domains of *C. trachomatis *MOMP and the known characteristics of antiparallel, amphipathic β-barrel strands in porins.

## Authors' contributions

HEF and HM carried out most of the experiments. HEF analysed and organised the data, and drafted the first version of the manuscript. RHA conceived the overall project, provided experimental guidance, carried out some of the experiments, and redrafted the manuscript. All the authors read and approved the final manuscript.

## Supplementary Material

Additional File 1 **Detergent extraction of recombinant MOMP. **Immunoblot with ECL detection following SDS-PAGE of OM proteins (10 μg per lane) from BL21 cells expressing OmpT-leadered *C. trachomatis *MOMP, induced for 2 hrs at 37°C. OM proteins (see Methods) were solubilised in 1% (w/v or v/v) octylglucoside, Triton X-100, Zwittergent 3–14 or LDAO, as indicated. NB & B are non-boiled and boiled samples, respectively. NE = non-expressing (control) cells. Click here for file

Additional File 2 **Optimisation of *C. trachomatis *MOMP expression and processing in BL21omp8 cells. **Growth curves of BL21omp8 cells expressing *C. trachomatis *MOMP with its native leader, in LB or SOC medium (means ± SEM, n = 4). Click here for file

Additional File 3 **MOMP epitopes are not unmasked by Tris buffer or EDTA in whole cell immunoblots. **Control BL21 cells and cells expressing "strand-deleted" constructs were suspended in 100 mM NaCl containing 50 mM Tris-HCl (pH 7.4) with or without 2 mM EDTA, applied to nitrocellulose membranes, and probed with anti-*C. trachomatis *MOMP polyclonal antibody. Click here for file

Additional File 4 **Recombinant MOMP does not form SDS-resistant oligomers. **SDS-PAGE and immunoblot analysis of *C. trachomatis *MOMP expressed with its native leader in BL21omp8 cells at 16°C (induced for 12 hrs in the presence of 0.1 mM IPTG). Lanes 1 & 2 contain 10 μg non-boiled and boiled OM proteins, respectively, solubilised in 1% (w/v) OG. Note successful transfer of high-MW proteins. Click here for file
