# Supplementary figures and images for: Surface expression, single-channel analysis and membrane topology of recombinant Chlamydia trachomatis Major Outer Membrane Protein
Source: BMC Microbiol. 2005 Jan 26;5:5. doi: 10.1186/1471-2180-5-5 (PMC549562; doi:10.1186/1471-2180-5-5)

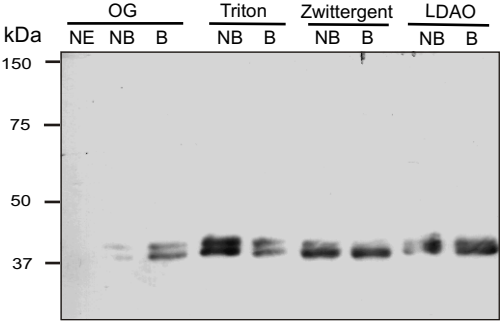

Supplement: Additional File 1 — Detergent extraction of recombinant MOMP. Immunoblot with ECL detection following SDS-PAGE of OM proteins (10 μg per lane) from BL21 cells expressing OmpT-leadered C. trachomatis MOMP, induced for 2 hrs at 37°C. OM proteins (see Methods) were solubilised in 1% (w/v or v/v) octylglucoside, Triton X-100, Zwittergent 3–14 or LDAO, as indicated. NB & B are non-boiled and boiled samples, respectively. NE = non-expressing (control) cells. [file 1471-2180-5-5-S1.pdf]

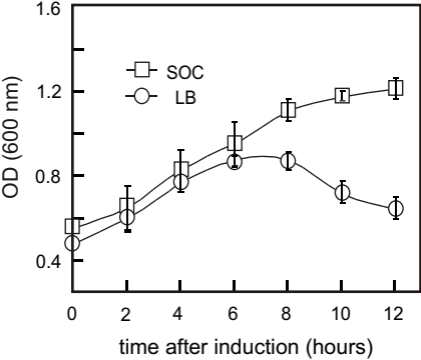

Supplement: Additional File 2 — Optimisation of C. trachomatis MOMP expression and processing in BL21omp8 cells. Growth curves of BL21omp8 cells expressing C. trachomatis MOMP with its native leader, in LB or SOC medium (means ± SEM, n = 4). [file 1471-2180-5-5-S2.pdf]

BL    mature    n-leader    delta5    delta5,6

Tris buffer

2 mM EDTA

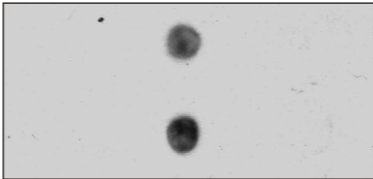

Supplement: Additional File 3 — MOMP epitopes are not unmasked by Tris buffer or EDTA in whole cell immunoblots. Control BL21 cells and cells expressing "strand-deleted" constructs were suspended in 100 mM NaCl containing 50 mM Tris-HCl (pH 7.4) with or without 2 mM EDTA, applied to nitrocellulose membranes, and probed with anti-C. trachomatis MOMP polyclonal antibody. [file 1471-2180-5-5-S3.pdf]

kDa

transfer

immunoblot

overlay

150

75

50

37

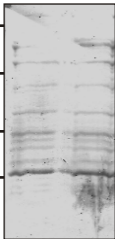

1

2

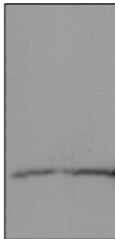

1

2

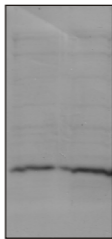

1

2

← MOMP

Supplement: Additional File 4 — Recombinant MOMP does not form SDS-resistant oligomers. SDS-PAGE and immunoblot analysis of C. trachomatis MOMP expressed with its native leader in BL21omp8 cells at 16°C (induced for 12 hrs in the presence of 0.1 mM IPTG). Lanes 1 & 2 contain 10 μg non-boiled and boiled OM proteins, respectively, solubilised in 1% (w/v) OG. Note successful transfer of high-MW proteins. [file 1471-2180-5-5-S4.pdf]
